# Severe Hemorrhagic Fever in Strain 13/N Guinea Pigs Infected with Lujo Virus

**DOI:** 10.1371/journal.pntd.0001801

**Published:** 2012-08-28

**Authors:** Brian H. Bird, Kimberly A. Dodd, Bobbie R. Erickson, César G. Albariño, Ayan K. Chakrabarti, Laura K. McMullan, Eric Bergeron, Ute Ströeher, Deborah Cannon, Brock Martin, JoAnn D. Coleman-McCray, Stuart T. Nichol, Christina F. Spiropoulou

**Affiliations:** 1 Viral Special Pathogens Branch, Division of High Consequence Pathogens and Pathology, Centers for Disease Control and Prevention, Atlanta, Georgia, United States of America; 2 School of Veterinary Medicine, University of California Davis, Davis, California, United States of America; University of Texas Medical Branch, United States of America

## Abstract

Lujo virus (LUJV) is a novel member of the *Arenaviridae* family that was first identified in 2008 after an outbreak of severe hemorrhagic fever (HF). In what was a small but rapidly progressing outbreak, this previously unknown virus was transmitted from the critically ill index patient to 4 attending healthcare workers. Four persons died during this outbreak, for a total case fatality of 80% (4/5). The suspected rodent source of the initial exposure to LUJV remains a mystery. Because of the ease of transmission, high case fatality, and novel nature of LUJV, we sought to establish an animal model of LUJV HF. Initial attempts in mice failed, but infection of inbred strain 13/N guinea pigs resulted in lethal disease. A total of 41 adult strain 13/N guinea pigs were infected with either wild-type LUJV or a full-length recombinant LUJV. Results demonstrated that strain 13/N guinea pigs provide an excellent model of severe and lethal LUJV HF that closely resembles what is known of the human disease. All infected animals experienced consistent weight loss (3–5% per day) and clinical illness characterized by ocular discharge, ruffled fur, hunched posture, and lethargy. Uniform lethality occurred by 11–16 days post-infection. All animals developed disseminated LUJV infection in various organs (liver, spleen, lung, and kidney), and leukopenia, lymphopenia, thrombocytopenia, coagulopathy, and elevated transaminase levels. Serial euthanasia studies revealed a temporal pattern of virus dissemination and increasing severity of disease, primarily targeting the liver, spleen, lungs, and lower gastrointestinal tract. Establishing an animal LUJV model is an important first step towards understanding the high pathogenicity of LUJV and developing vaccines and antiviral therapeutic drugs for this highly transmissible and lethal emerging pathogen.

## Author Summary

The pathogenic arenaviruses are a diverse group of human pathogens capable of causing a wide range of human illness ranging from encephalitis to severe hemorrhagic fever throughout the New and Old World. In 2008, a previously unknown virus (now named Lujo virus) caused a high case fatality outbreak (80%) in southern Africa. Limited data available from these patients indicated that LUJV HF was characterized by thrombocytopenia, elevated liver transaminases, coagulopathy, viral antigen in multiple tissues, neurological symptoms in some cases, and eventual death. The source of exposure of the index patient remains unknown. Due to the unusually high lethality and rapid human to human spread, we sought to develop an animal model of Lujo hemorrhagic fever. We report here that after infection with Lujo virus, Strain 13/N guinea pigs develop a hemorrhagic fever syndrome similar to the disease observed in human patients. This animal model of severe Lujo hemorrhagic fever is a critical first step to increase our understanding of this highly pathogenic virus, and to develop anti-viral therapeutics or experimental vaccines for this new and unique threat to human health.

## Introduction

Beginning in the 1930s, novel pathogenic arenaviruses have been increasingly recognized as emerging threats to human health [1], [2], [3], [4], [5], [6], [7], [8], [9], [10]. During the 1960s and 1970s, several previously unknown arenaviruses emerged as a significant public health threats and causes of a severe and often fatal human hemorrhagic fever (HF) syndrome. In 2008, Lujo virus (LUJV), a novel member of the family *Arenaviridae*, was first identified after an outbreak of severe HF in southern Africa [11]. During this outbreak, the index patient was transported by air from **Lu**saka, Zambia, to a private hospital in **Jo**hannesburg, South Africa, thus giving the virus its name, **Lu**-**Jo**.

The index patient died from the infection approximately 12 days after the onset of the presumed first symptoms, and 2 days after hospitalization in Johannesburg. During transport and hospitalization of the index patient, a total of 4 health care workers (3 nurses and 1 janitor) were infected with LUJV. After a period of 10–13 days of progressively severe illness, 3 of these individuals died, resulting in a total case fatality of 80% (4/5). Limited data available from these patients indicated that LUJV HF was characterized by thrombocytopenia, elevated liver transaminases, coagulopathy, viral antigen in multiple tissues, neurological symptoms in some cases, and eventual death. While the outbreak was small, the ease with which LUJV spread among the primary, secondary, and tertiary contacts with the index patient, and the lack of a defined etiology, caused significant alarm. The viral cause of the outbreak was identified as a novel arenavirus only after the last case fatality [12]. The suspected source of exposure of the index patient to LUJV (presumably a rodent) remains unknown.

The arenaviruses are a large and genetically diverse group of over 30 viruses broadly divided into New World and Old World serogroups. They are exclusively rodent-borne, except Tacaribe virus, which was isolated from a bat [13], [14]. Phylogenetically, LUJV is distinct from both the Old World and New World arenavirus lineages, and is the sole member of a distinct branch more closely related to the known Old World arenaviruses [12]. Although many arenaviruses are not pathogenic, a large number can cause a spectrum of human disease ranging from neurological symptoms in pediatric or immunocompromised patients (e.g., Lymphocytic choriomeningitis virus, LCMV) [15], [16], [17], [18], [19], [20], to hemorrhagic syndromes with high case fatalities (e.g., Lassa virus (LASV), Junin virus (JUNV), and Machupo, Guanarito, Chapare, and Sabia viruses) [21], [22], [23].

All arenaviruses are enveloped particles containing bi-segmented, single-stranded, ambi-sense RNA genomes encoding a total of 4 genes [13]. The large (L) genome segment (∼7.2 kb) contains the viral RNA-dependent RNA polymerase and the multi-functional Z-protein, an important matrix protein that is also responsible for virus budding. The small (S) genome segment (3.4 kb) encodes the viral nucleoprotein (NP) and the glycoprotein precursor (GPC), which is post-translationally processed into the G1 and G2 structural proteins. Both NP and Z proteins function as viral virulence factors antagonizing host cell interferon responses by a variety of mechanisms [21], [24], [25], [26].

Although many experimental vaccine candidates and antiviral drugs are under development [27], [28], [29], [30], currently the only available vaccine for any pathogenic arenavirus is the live-attenuated Candid1 vaccine for JUNV, which is restricted for use only in high-risk individuals such as laboratorians and those living in endemic areas [31], [32]. The antiviral drug ribavirin has shown some efficacy in treating LASV and some other arenaviruses, but its side effects limit use to high-risk exposures and severe cases [33], [34]. Due to the unique characteristics of the LUJV outbreak and the highly novel genomic nature of LUJV, we sought to establish an animal model capable of developing HF similar to that observed in the 4 fatal human cases. Establishing a robust animal model is necessary for further investigating LUJV pathogenesis, and to provide a system to test potential vaccine candidates and antiviral therapeutic drugs. Initial experiments with LUJV infection in 2-day-old newborn and 14-day-old weanling mice failed to provide a lethal model. This was highly surprising given the near uniform lethality of pathogenic New World or Old World arenaviruses in newborn or weanling mice, respectively [12], and highlights another unique feature of LUJV.

We next attempted to develop a LUJV HF model in guinea pigs (*Cavia porcellus*), which have been used since the 1960s as reliable models for a variety of pathogenic New World and Old World arenaviruses [35], [36], [37], [38], [39], [40], [41], [42], [43], [44]. The inbred strain 13/N guinea pig is highly susceptible to LASV infection; the animals develop severe, progressive, and ultimately fatal disease 15–21 days post-infection (PI), showing many pathological changes that closely mimic Lassa fever in humans [37]. Given their susceptibility to Old World arenaviruses, we began experiments in strain 13/N guinea pigs to study LUJV virulence and pathogenesis. Here, we report successfully establishing a lethal model of LUJV HF in strain 13/N guinea pigs infected with either authentic wild-type or a recombinant full-length reverse genetics-derived LUJV. This robust and highly uniform animal model will permit further detailed investigations into the molecular determinants of LUJV pathogenesis, and provide an *in vivo* system for testing novel anti-viral therapeutics and vaccines against this highly pathogenic and unique arenavirus.

## Materials and Methods

### Biosafety

All work with infectious virus or infected animals was conducted at the Centers for Disease Control and Prevention (CDC, Atlanta, Georgia, USA), in a biosafety level 4 laboratory. All laboratorians and animal handlers adhered to international biosafety practices appropriate for biosafety level 4, strictly following infection control practices to prevent cross-contamination between individual animals. All animals were individually housed in an isolator-caging system (Thoren Caging, Inc., Hazleton, PA, USA) with a HEPA-filtered inlet and exhaust air supply.

### Ethics statement and animal husbandry

All procedures and experiments described herein were approved by the CDC Institutional Animal Care and Use Committee (IACUC) and conducted in strict accordance with the Guide for the Care and Use of Laboratory Animals [45]. All animals were housed in a climate-controlled laboratory with a 12 h day/12 h night cycle. The CDC is an Association for Assessment and Accreditation of Laboratory Animal Care International (AAALAC) fully accredited research facility. No human patient derived clinical materials were used in the completion of these studies.

### Mice

A total of 8 litters of pregnant outbred mice were obtained from a commercial vendor (Charles River Laboratories, Wilmington, MA, USA). All mice were housed as individual family units, and supplied a commercially available mouse chow and water *ad libitum*. The cage environment was enriched with large amounts of soft bedding, shredded paper, and cotton nestlets. After infection, each animal was observed at least once per day, and its health assessed and scored by experienced CDC veterinarians or animal health technicians. Animals were humanely euthanized with isoflurane vapors once clinical illness scores (including, but not limited to, neurological signs, piloerection, ocular discharge, weight loss, changes in mentation, ataxia, dehydration, or dyspnea) indicated that the animal was in distress or in the terminal stages of disease.

### Guinea pigs

A total of 47 strain 13/N guinea pigs (healthy adult males and females aged 1.0–1.5 years) were obtained from an established breeding colony located at the University of Iowa (Ames, IA, USA). All animals were housed individually on deep soft bedding and given food (commercial guinea pig chow, alfalfa cubes, and fresh green parsley), and water supplemented with guinea pig appropriate vitamins *ad libitum*, following standard laboratory animal husbandry protocols for guinea pigs. After infection, each animal was observed at least twice per day, and its health assessed and scored by experienced CDC veterinarians or animal health technicians. Animals were humanely euthanized with isoflurane vapors and sodium pentobarbital (Schering-Plough, Kenilworth, NJ, USA) at either predetermined times PI, or once clinical illness scores (including, but not limited to, piloerection, ocular discharge, weight loss, changes in mentation, ataxia, dehydration, dyspnea, or hypothermia) indicated that the animal was in the terminal stages of disease.

### Viruses

Wild-type LUJV (wtLUJV) from the Centers for Disease Control and Prevention Viral Special Pathogens Branch reference collection was passaged in VERO-E6 cells five times before use. A full-length recombinant LUJV (recLUJV) was derived from cDNA plasmids using T7-driven reverse genetics as reported in Bergeron et al. (*manuscript in review*). To differentiate recLUJV from wtLUJV, a few silent (non-coding) nucleotide changes were introduced into the full-length L segment clones of recLUJV (also described in Bergeron et al., *manuscript in review*). For mouse experiments, wtLASV-Josiah and wtJUNV-XJ13 were prepared and utilized as described in [46], [47]. Prior to use, all virus stocks were titrated and full-length genomic sequences were verified using standard techniques as described in [46], [47].

### Animal infection procedures

#### Mice

For infection, groups of 10 animals (2 or 14 days old) were inoculated intracranially with 500 focus-forming units (FFU) of wtLUJV, wtLASV-Josiah, or wtJUNV-XJ13 in 10 uL of Dulbecco's modified Eagle's medium (DMEM; Invitrogen Corp., Grand Island, NY, USA) using a 0.3 mL 29-gauge tuberculin syringe, or with up to 2.0×10^3^ FFU of wtLUJV by either subcutaneous or intraperitoneal routes following techniques described in [46], [47].

#### Guinea pigs

For infection, all animals were briefly anesthetized with isoflurane vapors and inoculated intraperitoneally in the lower right quadrant using a 25-gauge 5/8 inch needle attached to a 1 mL syringe. All animals were inoculated with 250 uL containing 1.0×10^5^ FFU of either wtLUJV (N = 28) or recLUJV (N = 13), or with sterile diluent (DMEM, Invitrogen; N = 6). After infection, all animals were weighed and rectal temperatures taken daily. Each animal was observed and its health assessed and scored by experienced CDC veterinarians or animal health technicians at least twice per day. The experiment was conducted in 2 phases: 1) to assess the overall lethality of wtLUJV or recLUJV infection, groups of 10 animals were infected and monitored at least twice daily until found dead or euthanized according to a predetermined euthanasia/clinical illness scoring algorithm indicating severe disease or moribundity; and 2) a serial euthanasia phase was conducted to more closely follow the kinetics of LUJV dissemination and pathology. To accomplish the second phase, groups of 3 animals were scheduled for euthanasia at 2, 5, 7, 9, 12, or 14 days PI with wtLUJV, and at day 9 PI with recLUJV. Due to greater than expected virulence of LUJV, the actual days of serial euthanasia and specimen collection were modified as follows: day 2, N = 3; day 5, N = 3; day 7, N = 3; day 9, N = 3 wt, N = 3 rec; day 12, N = 3; day 14, N = 2. Two moribund animals from the serial euthanasia phase were euthanized on day 11 and were considered as terminal cases; the data from these animals was combined with data collected from the first phase of the experiment. The sham-inoculated control animals (N = 6) were euthanized at the end of the study, approximately 21 days post-inoculation.

### Total RNA extraction

Specimens of liver, lung, spleen, kidney, whole blood, urine, and/or pleural effusion or abdominal fluid (if present) were collected sterilely at 2, 5, 7, 9, 12, and 14 days PI, and from moribund animals that reached experimental end-points. For RNA extraction, approximately 100 mg specimens of tissues were stored in RNA extraction buffer (Tripure, Roche Diagnostics, Indianapolis, IN, USA) at −80°C until homogenization in a high-throughput tissue grinder (Genogrinder2000, BT&C Inc., Lebanon, NJ, USA). An equal volume of molecular grade chloroform was added to each specimen homogenate and vortexed. After a 10 minute spin at >10,000 rpm in a microcentrifuge, the supernatant was collected and an equal volume of 70% ethanol was added. The supernatant and ethanol was used for total RNA extraction (RNAeasy 96 platform, Qiagen, Valencia, CA, USA) following the manufacturer's recommended protocols.

### Viremia testing by quantitative reverse transcription PCR (qRT-PCR)

Briefly, LUJV RNA was detected using qRT-PCR with primers and probe with internal (Zen, Integrated DNA Technologies) and 3′ Iowa Black-FQ quencher moieties specific for the NP gene (forward primer: 5′-CTCACACCCACAGGAAAT-3′; reverse primer: 5′-GGCCATACATCTCTTCCAGA-3′; probe: 5′-6FAM-ACCCTACAC/Zen/CTCCACAGAACGAAAG-IowaBlackQuencherFQ-3′). For each viral genome detection reaction, 1 uL of total RNA was added to a one-step qRT-PCR (Invitrogen), where the first stand was synthesized using Superscript III at 50°C for 15 min, denatured at 94°C for 2 min, and amplified for 40 cycles of 94°C for 15 s and 60°C for 1 min (ABI 7500, Life Sciences, Grand Island, NY, USA). LUJV RNA genome equivalents in infected blood, fluid, or tissue specimens were quantitated using a standard curve generated by serial dilutions of a known-titer stock virus spiked into normal whole guinea pig blood. The results of all qRT-PCR tests were normalized to endogenous rodent-specific controls (glyceraldehyde 3-phosphate dehydrogenase (GAPDH), Invitrogen) following the manufacturer's recommended protocols to account for sample-to-sample variation in RNA extraction efficiency.

### Hematological parameters

Guinea pig whole blood was collected by intracardiac techniques into either EDTA-coated or heparin-coated vacutainer tubes. Complete blood counts (CBC) were obtained using the Hematrue blood analyzer (HESKA, Loveland, CO, USA). Blood chemistry profiles were obtained from heparinized samples using either the Piccolo point of care chemistry analyzer (Abaxis, Union City, CA, USA) or the Hitachi P-module analyzer (Hitachi Hi-Tech, Tokyo, Japan).

### Tissue-specific gene induction qRT-PCR assays

Liver, spleen, lung, and kidney tissues were collected 2, 5, 7, 9, 12, and 14 days PI (serial euthanasia groups), and from moribund animals that reached experimental end-points of terminal disease. Specimen RNA was treated with DNase I (Qiagen) followed by RNA cleanup utilizing the RNeasy Mini columns and wash buffers (Qiagen) per manufacturer's recommendations. Total RNA was quantified after DNase I treatment and cleanup using a NanoDrop spectrophotometer (Thermo Scientific, Wilmington, DE, USA). Previously reported gene specific primers were used to detect interleukin (IL)-1b, IL-2, IL-8, IL-12p40, tumor necrosis factor alpha (TNFa), transforming growth factor beta (TGFb), regulated upon activation normal T-cell expressed and secreted (RANTES), interferon gamma (IFNg), monocyte chemotactic protein (MCP)-1, inducible nitric oxide synthetase (iNOS), and GAPDH [48], [49]. Generally, 50 ng of RNA was used for each individual qRT-PCR; however, for samples with low RNA concentrations, a minimum of 5 ng was used. Invitrogen's SuperScript III Platinum SYBR green one-step qRT-PCR kit was used for 25 uL total volume reactions, with final reaction concentrations of 1× reaction mix, 0.2 M primers, 0.5 uL enzyme mix, and 50 ng RNA. Identical thermocycling profiles were utilized for all assays; 55°C for 10 min; 95°C for 5 min; amplification for 40 cycles of 95°C for 15 s and 58°C for 30 s; 40°C for 1 min; and a dissociation curve (CFX96 Touch, Bio-Rad, Hercules, CA, USA). A gene-specific real-time assay was developed for IL-10 (GenBank accession JN020146) using the GenScript real-time PCR primer design tool, IL10 F 5′-CACAGGATCAGCTGGACAAC–3′, IL10 R 5′-GGGCATCACCTCCACTAGAT-3′, and IL10 Probe 5′(FAM)-CCTGGGTTGCCAAGCCTTGTC-(BHQ1)3′. The Invitrogen SuperScript III Platinum one-step qRT-PCR kit was used for 25 µL total volume reactions following the manufacturer's protocol and the following thermocycling profile; 55°C for 10 min, 95°C for 2 min, amplification for 40 cycles of 95°C for 15 s and 60°C for 30 s. Guinea pig GAPDH was used as the internal control calibrator. Bio-Rad CFX manager software v2.1 was used to analyze the cycling threshold (C_T_) values and melt curves for each reaction, and results were analyzed using the comparative C_T_ method as described by Schmittgen and Livak [50]. The fold change of each serial euthanasia group was compared to mean fold change of the sham-infected control guinea pigs (N = 6), and the standard error of the mean was calculated for each experimental group. Due to technical issues during RNA extraction, the day 5 PI spleen data was generated from only 1 infected animal.

### Histology

At the time of collection, tissue specimens were fixed in 10% neutral buffered formalin and gamma-irradiated (2.0×10^6^ RAD) prior to sectioning into 4 um-thick slices and staining with hematoxylin and eosin following routine histology protocols.

### Statistical analyses

All analyses were completed using the PRISM v5.0 program (Graphpad, LaJolla, CA, USA). Potentially significant differences between wtLUJV and recLUJV groups were evaluated using a student's t-test. In subsequent analyses, wtLUJV and recLUJV data were combined for all day 9 and terminal group analyses. For the complete blood counts, clinical chemistry, and gene regulation data, significant differences between LUJV-infected and sham-infected animals at each time point were analyzed using a one-way analysis of variance (ANOVA) with Dunnett's adjustment for multiple comparisons (*p<0.05; **p<0.01, ***p<0.001).

## Results

### LUJV is not virulent in 2-day-old newborn or 14-day-old weanling mice

Mice were monitored for 28 days PI with 500 FFU of wtLUJV, wtLASV-Josiah, wtJUNV-XJ13, or inoculation with DMEM as a negative control. As expected, wtJUNV-XJ13 caused uniform neurological signs and lethality by 15 days PI in 2-day-old, but not 14-day-old mice. In contrast and as expected, wtLASV infection resulted in near uniform (90%) lethality in 14-day-old weanling mice, but was non-lethal in 2-day-old newborn mice. Infection was confirmed by the detection of anti-Lujo virus specific antibodies at 28 days post-infection in 3 surviving animals. Surprisingly, wtLUJV did not cause any signs of clinical illness or lethality in either 2-day-old or 14-day-old mice regardless of the dose (up to 2.0×10^3^ FFU) or inoculation route (intracranial, subcutaneous, or intraperitoneal) (Fig. S1 and data not shown).

### LUJV causes severe disease and lethality in adult male and female strain 13/N guinea pigs

#### Clinical presentation

**Figure 1:**
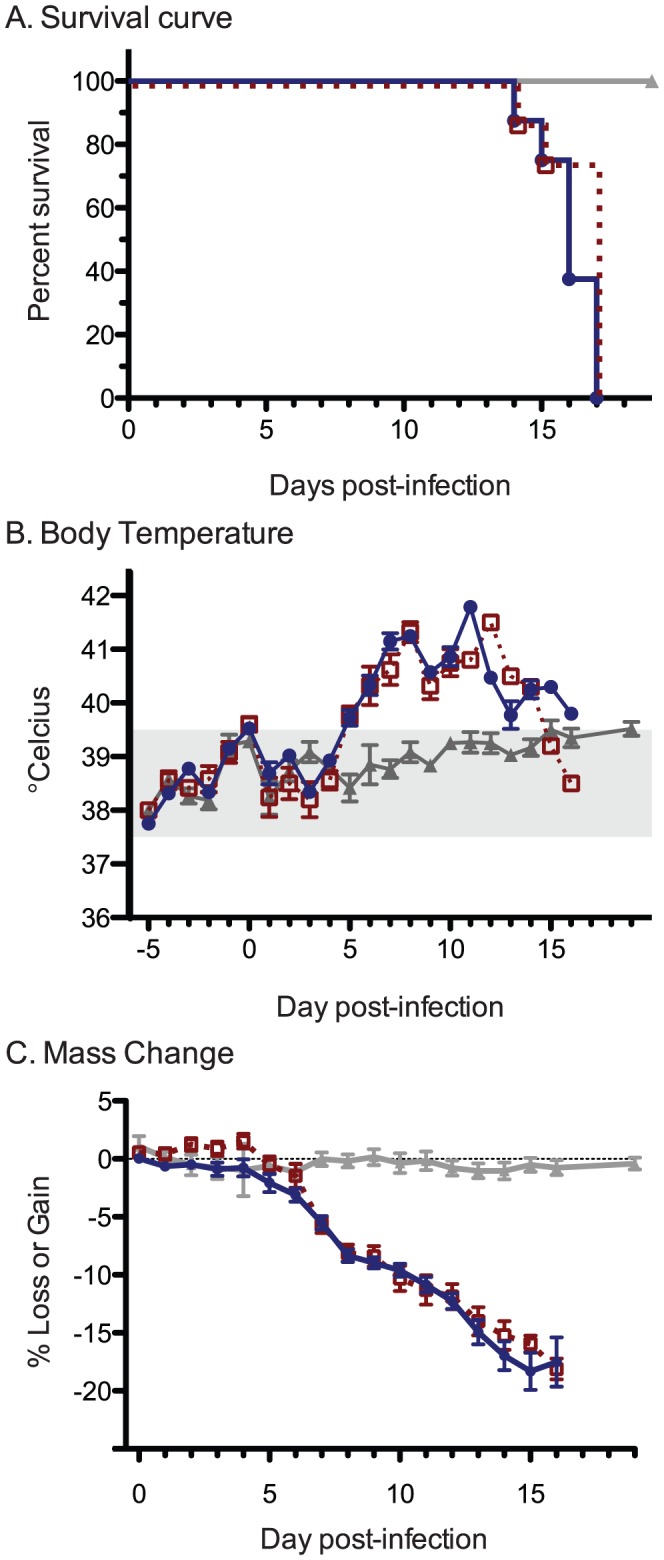
Clinical outcomes. A. Survival times of guinea pigs inoculated with either wild-type Lujo virus (wtLUJV), recombinant LUJV (recLUJV), or sham DMEM controls. Mean survival times of animals infected with wtLUJV and recLUJV were not significantly different. B. Body temperatures (rectal) depicted by group per day post-infection (PI) or post-sham inoculation. Groupwise means ±1 standard error of the mean (SEM) are depicted. C. Percent (%) change in body mass by group per day PI or after sham inoculation. In all panels, asterisks depict the day of the onset of detectable clinical signs (ocular discharge). All data were normalized to an average starting weight days −5 to 0 pre-infection. wtLUJV = dark blue circle; recLUJV = open red box; sham controls = closed grey triangle.

All animals infected with either the wtLUJV (N = 28 total; full-duration disease monitoring group N = 10; serial euthanasia group N = 18) or with recLUJV (N = 13 total; full-duration disease monitoring group N = 10; serial euthanasia group N = 3) experienced an onset of progressive illness starting approximately 5 days PI. The illness was characterized clinically by pyrexia, loss of body weight, bilateral, encrusted, light tan-colored ocular discharge, dehydration (as demonstrated by sunken eyes and tacky mucous membranes), ruffled fur, piloerection, lethargy, hematuria, frank external genitourinary hemorrhage (in 1 animal), and eventual moribundity followed rapidly by death (Fig. 1A). Animals were either found dead (wtLUJV N = 1; recLUJV N = 2) or euthanized when moribund (wtLUJV N = 9; recLUJV N = 8) 11–16 days PI (Fig. 1A). No sham-infected control animals (N = 6) developed any signs of clinical illness during the duration of the experiment.

#### Body temperatures

Starting at 5–6 days PI with both wtLUJV or recLUJV, the animals uniformly developed a febrile response ranging from a mean of 39.6°C on day 5 PI to a peak of 42.1°C on day 11 PI (Fig. 1B). Individual animal temperatures often exceeded 42.0°C on days 11 and 12 PI (data not shown). The febrile response was maintained until immediately prior to moribundity and death, when body temperatures rapidly declined to values below normal (data not shown). As a group, the sham-infected animals maintained body temperatures in the normal range (37.5–39.5°C).

#### Weight loss

By day 6 PI, the majority of LUJV-infected animals began to lose weight, with all animals demonstrating at least 5% body mass loss by day 7 PI (Fig. 1C). Individual animal weight loss ranged from 4 to 5% per day until found moribund or dead by 15 days PI. The weight loss in several animals appeared to follow a biphasic pattern of 10–15% loss 5–8 days PI, a period of moderate stabilization for 2–3 days, and a final decline in body weight 12–15 days PI (Fig. 1C). All sham-infected controls maintained body weight within ±5% of starting weight throughout the duration of the experiment.

#### Viral loads in tissues and fluids

**Figure 2:**
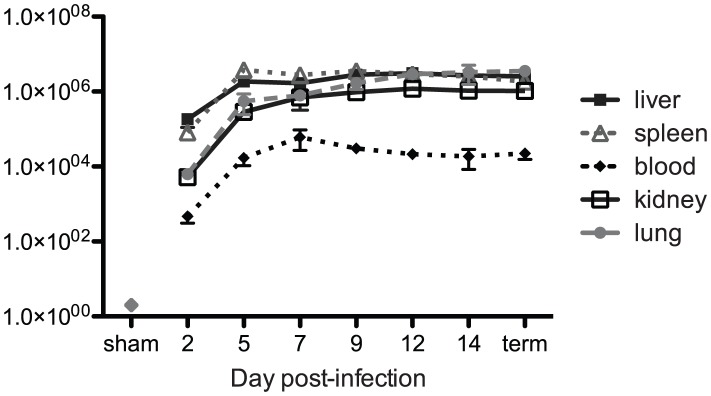
Viral RNA load. Viral RNA loads (qRT-PCR-derived tissue culture infective dose 50 (TCID_50_) equivalents) in blood, liver, spleen, lungs, and kidney are shown.

Within 48 h PI, a dramatic rise in viral load, measured by qRT-PCR and reported as tissue culture infective dose 50 (TCID_50_) equivalents (eq), was detected in all tissues and fluids analyzed (whole blood: 4.64×10^2^ eq; liver: 1.83×10^5^ eq; spleen: 8.13×10^4^ eq; lung: 6.22×10^3^ eq; kidney: 5.24×10^3^ eq; urine: 2.20×10^3^ eq) (Fig. 2). Over the duration of the experiment, a temporal pattern emerged, suggesting the liver and spleen as initial sites of high-level viral replication (2–9 days PI), with increasing amounts of virus detected in lungs and kidneys 12 and 14 days PI, and in all terminal cases exceeding 1.0×10^6^ eq. Overall, wtLUJV and recLUJV rapidly caused disseminated infection of various visceral organs, with high viral loads detected throughout the experiment. Viral loads in blood, abdominal fluid, and pleural effusions collected from terminally ill animals were also positive for viral RNA, with titers ranging from 2.98×10^4^ TCID_50_ eq to >1.0×10^5^ TCID_50_ eq by day 5 and throughout the remainder of the experiment.

#### Hematology

**Figure 3:**
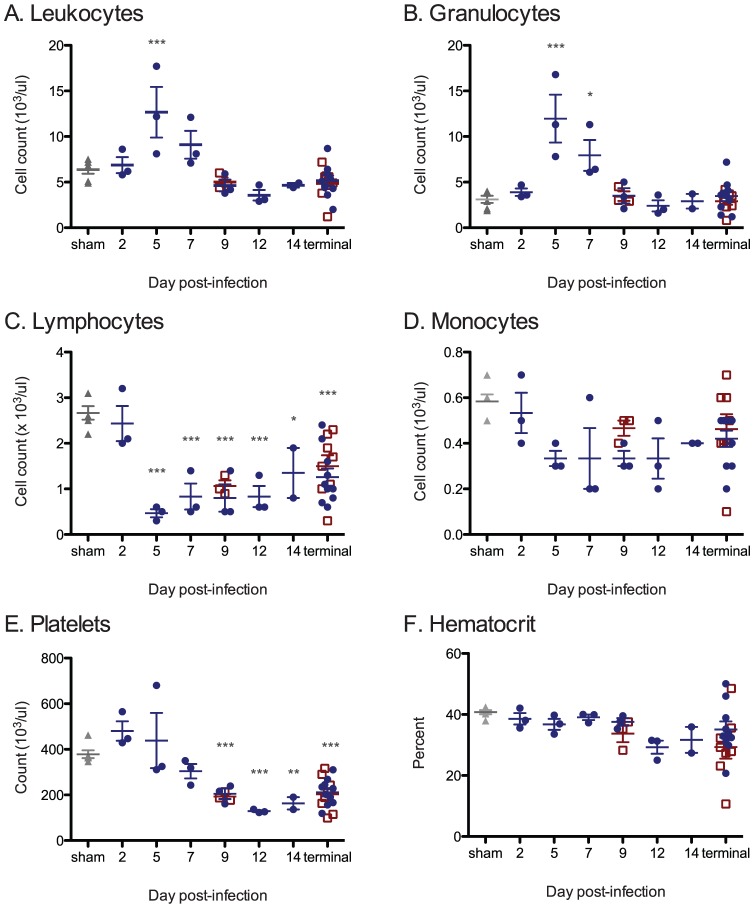
Complete blood counts. Individual animal blood cell parameters. In all panels, each animal's data are depicted along with the groupwise mean (horizontal bar) ±1 SEM (error bars). Panel A. Leukocytes, B. Granulocytes, C. Lymphocytes, D. Monocytes, E. Platelets, and F. Hematocrit are depicted. Significant deviations from normal control animal values are indicated by asterisks (p-value: *<0.05; ***<0.001). wtLUJV = dark blue circle; recLUJV = open red box; sham controls = closed grey triangle.

Automated differential complete blood counts were conducted on groups of 3 animals at days 2, 5, 7, 9, 12, or 14 PI with wtLUJV; day 9 PI with recLUJV; and on moribund animals (N = 9 wtLUJV; N = 8 recLUJV) (Fig. 3). No significant differences were detected between animals infected with wtLUJV or recLUJV (Fig. 3 and data not shown); therefore all data from wtLUJV and recLUJV animals were combined. We observed a transient but marked left-shift inflammatory response starting at day 5 PI and continuing through day 7 PI. This response was characterized by a significant marked leukocytosis (Fig. 3A; mean 12.6×10^3^ uL^−1^; reference range (REF) = 9.9×10^3^ uL^−1^±30%), and a significant marked non-degenerative granulocytosis (Fig. 3C; mean 11.9×10^3^ uL^−1^; REF = 3.1×10^3^ uL^−1^±50%). By 9–14 days PI, the animals developed a mild leukopenia that persisted until moribundity. A moderate to marked lymphopenia was observed starting on day 5 PI (Fig. 3B, p<0.001; mean 0.4×10^3^ uL^−1^; REF = 2.7×10^3^ uL^−1^±30%) and continuing for the duration of the experiment. Differential counts revealed a shift in lymphocyte/neutrophil percentages from 46.7%/43.8% in normal control animals to an observed maximum shift of 5.5%/92.3% 7 days PI (data not shown). Mild but non-significant (p>0.05) decreases in monocytes were observed during the course of the experiment (Fig. 3D). During the initial stages of infection, platelets were mildly elevated within the expected normal range 2, 5, and 7 days PI (Fig. 3E; REF = 250–850×10^3^ uL^−1^). However, by 12 and 14 days PI, and in terminal stage animals, moderate to marked thrombocytopenia was observed (Fig. 3E; ranging 99–163×10^6^ uL^−1^). During the course of infection, we observed mild anemia with concomitant declines in hematocrit (Fig. 3F), hemoglobin, and total red blood cells (data not shown). The most severely affected animal was euthanized on day 16 PI with massive frank hemorrhage in the peritoneal cavity, pan-leukopenia (total white blood cell count = 3.8×10^3^ uL^−1^), marked thrombocytopenia (99×10^3^ uL^−1^), and profound anemia with a hematocrit of 10.1% (REF = 43±12%).

#### Blood chemistry

**Figure 4:**
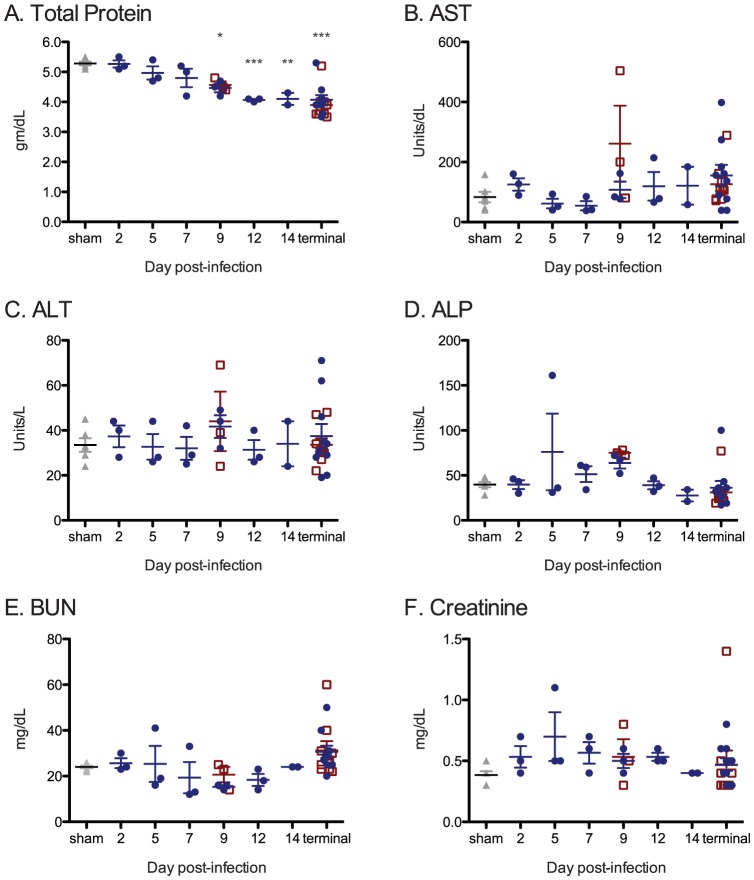
Comprehensive serum chemistry analyzes. A. Total Protein, B. Aspartate transferase (AST), C. Alanine transferase (ALT), D. Alkaline phosphatase (ALP), E. Blood urea nitrogen (BUN) and F. Creatinine. In all panels, each animal's data are depicted along with the groupwise mean (horizontal bar) ±1 SEM (error bars). Significant deviations from normal control animal values are indicated by asterisks (p-value: *<0.05; **<0.01; ***<0.001). wtLUJV = dark blue circle; recLUJV = open red box; sham controls = closed grey triangle.

Comprehensive blood chemistry analyses were completed using lithium-heparinized whole blood (Fig. 4). Electrolytes (sodium, potassium, chloride, and calcium) levels remained within the normal reference ranges at all timepoints regardless of the day PI or virus (wtLUJV or recLUJV) used. In moribund animals euthanized at the terminal stages of disease, a mild but significant hypoproteinemia (Fig. 4A; mean 4.0 g/dL; REF = 4.5–5.9 g/dL) and hypoalbuminemia (data not shown; mean 1.9 g/dL; REF = 2.3–3.0 g/dL) were observed. A consistent but non-significant and mild elevation in tissue transaminases (aspartate transferase, alkaline phosphatase, and alanine transferase; REF<60 IU/L) was detected late in the course of the disease, with levels highest on day 9 PI (Fig. 4B–D). Normal to moderate elevations of blood-urea nitrogen (approaching 60 mg/dL; REF = 15.7–31.5 mg/dL), and very mild or no elevation in creatinine levels (mean 0.5 mg/dL; REF<0.5 mg/dL) were observed, consistent with pre-renal azotemia, dehydration, extravascular hemolysis, and/or intra-intestinal hemorrhage.

#### Necropsy anatomical findings

**Figure 5:**
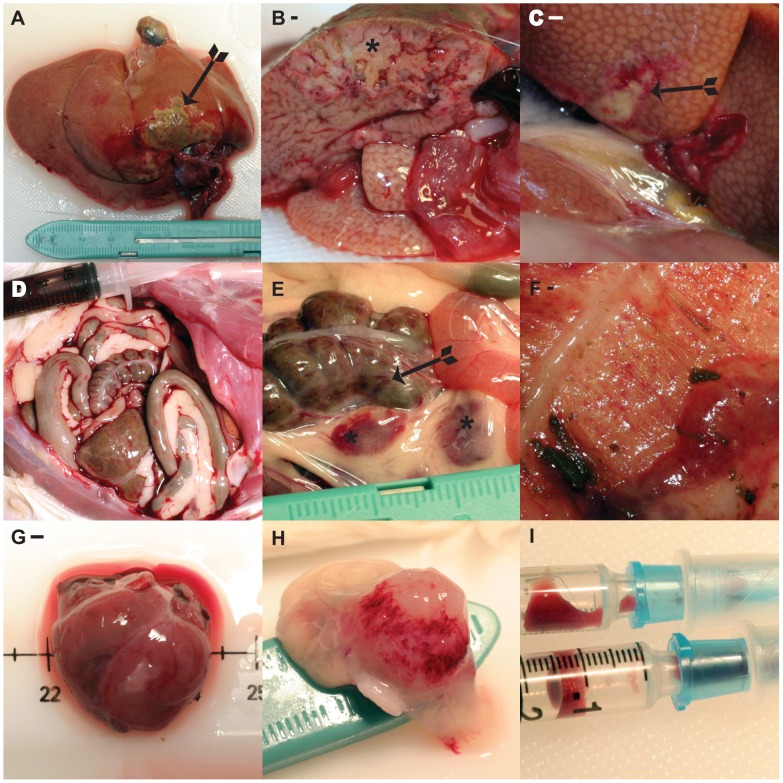
Necropsy gross anatomical findings. A. Whole liver from terminally infected animal. Note the marked congestion and extensive pan-lobular zone of hepatic damage due to infarction near the liver hilus that is surrounded by a well-demarcated hyperemic rim of hepatic parenchyma (arrow). B. Cross-section of the liver in panel A at the level of the main portal vein through the center of a zone of hepatic necrosis and infarction (asterisk). C. Another example of the severe hepatic congestion, necrosis, and infarction observed in all terminal cases (arrow). D. Frank hemorrhage (>35 mL) in the abdominal cavity. E. Severely congested and hemorrhagic mesenteric lymph nodes (asterisks), and severe congestion and hemorrhage on the serosal surface of the cecum (arrows). F. Mucosal surface of the cecum in panel E; note the severe congestion and extensive petechiation. G. Example of the severe dilated cardiomyopathy observed in the majority of infected animals beginning at approximately day 12 PI. H. Serosal surface of the bladder; note the severe petechiation. I. Contents of the bladder in panel H, consistent with hematuria and frank hemorrhage collected *in situ* by cystocentesis. Panels A, B, C, G, H (wtLUJV infected). Panels D, E, F (recLUJV infected).

No animal displayed overt signs of gross tissue pathology until 7 days PI. Starting at day 7 PI, liver tissues became progressively more pale and friable, and demonstrated enhanced reticular patterns of reddening suggestive of locally extensive to diffuse hepatic congestion; these signs persisted through day 14 PI or to the point of moribundity or death (Fig. 5A). By 11–12 days PI, we observed well-demarcated, multifocal pale foci with a hyperemic rim of hepatic parenchyma. On or after day 14 PI, we visualized large, locally extensive pan-lobular areas of pale, grossly necrotic tissue with a hyperemic rim of tissue, often adjacent to portal veins (Figs. 5B and 5C). Strikingly, starting 5 days PI and continuing through the course of the disease, animals developed accumulations of abdominal fluid (ascites) ranging in character from 1.0–2.0 mL of clear to pale yellow transudate on day 5, to moderate volumes (5.0–9.0 mL) of serosanguineous fluid 9–12 days PI, to frank blood that, in the most extreme example, exceeded 35.0 mL on day 16 PI (Fig. 5D). Mesenteric, ileocecocolic, and mediastinal lymph nodes became severely congested and hemorrhagic by 9 days PI (Fig. 5E). Generally, by day 9 PI and all subsequent time points, distention of the small bowel (jejunum), and evidence of hemorrhage on the mucosal and serosal surfaces of the large bowel (distal cecum) were observed (Fig. 5F). Starting on day 12 PI, approximately 50% of the animals had moderately to severely enlarged and flaccid hearts with thinning of the right ventricular wall. The most severely affected animals had a severely distended right ventricle wall grossly consistent with dilated cardiomyopathy (Fig. 5G). Signs of gross kidney pathology were observed after 12 days PI as a hyperemic rim at the junction of the renal cortex and medullary junction. In most cases, by day 14 PI and in terminal animals, the bladder of infected animals was filled with a dark fluid consistent with hematuria and contained multiple petechia on the serosal and mucosal surface (Figs. 5H and 5I). Consistently, animals in the terminal stages of LUJV induced disease developed multiple gross anatomical signs of profound coagulopathy suggestive of HF and bleeding diatheses.

#### Histological findings

**Figure 6:**
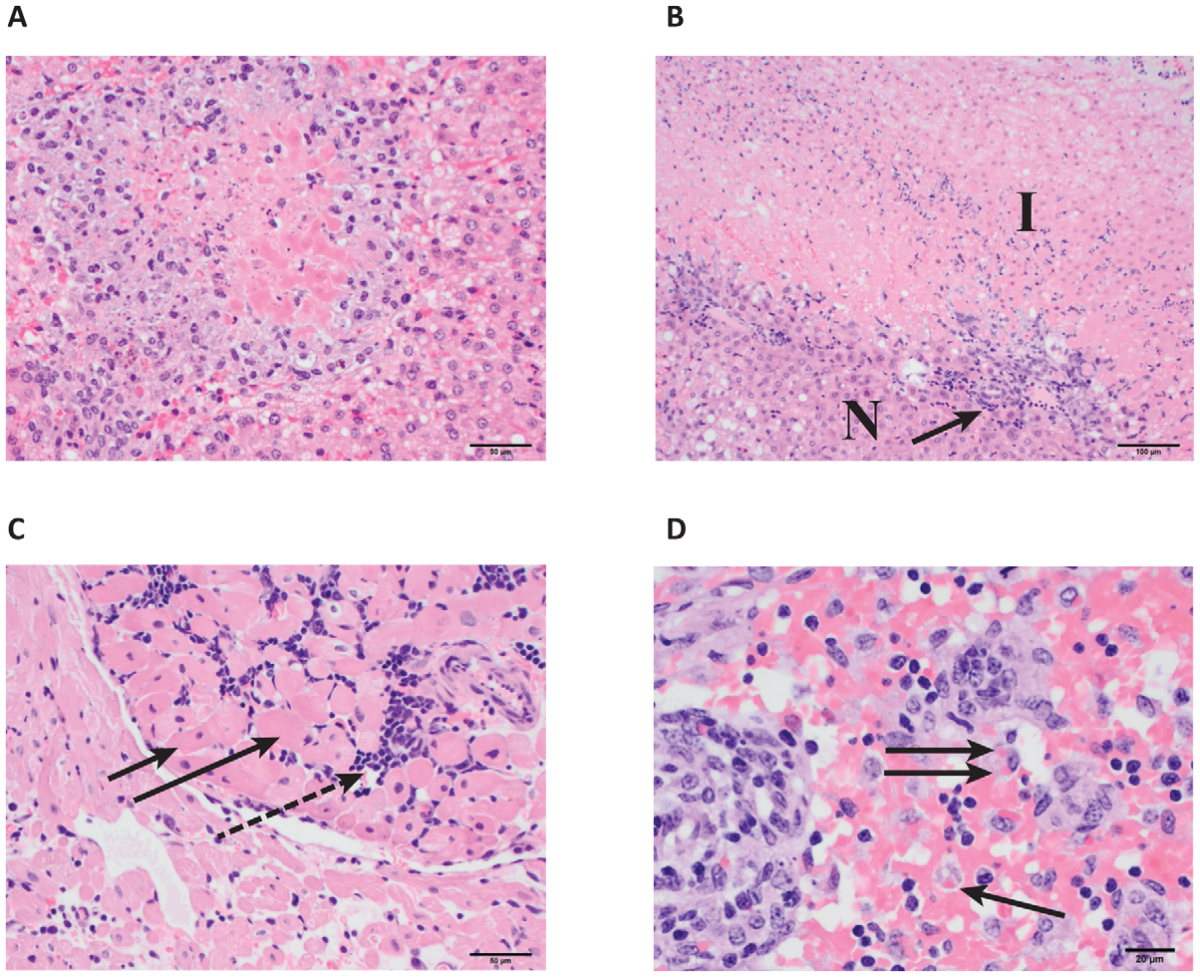
Histological findings. A. High-magnification photomicrograph of a liver from a guinea pig infected with LUJV. The center is composed of necrotic hepatocytes admixed with cellular debris, and is surrounded by moderate numbers of histiocytes. Hematoxylin and eosin stain; 200× original magnification; scale bar = 50 µm. B. Low magnification photomicrograph of the interface of normal (N) and infarcted (I) area of the liver from a guinea pig with LUJV infection. The infarcted area is cell-poor, and inflammatory cell reaction (arrow) is observed at the interface. Hematoxylin and eosin stain; 100× original magnification; scale bar = 100 µm. C. High-magnification photomicrograph of the papillary muscles of the heart from a guinea pig with LUJV infection. The muscle fibers (solid arrows) are slightly swollen and hypereosinophilic. Small nests of mononuclear inflammatory cells (dashed arrow) separate the myofibers. Hematoxylin and eosin stain; 200× original magnification; scale bar = 50 µm. D. High magnification photomicrograph of the mesenteric lymph-node from a guinea pig infected with Lujo virus (terminal case). The medullary sinuses are filled with large numbers of erythrocytes and there are scattered macrophages that contain intracytoplasmic erythrocytes (erythrophagocytosis, arrows). This lesion is compatible with a prior hemorrhage in the drainage area of the mesenteric lymph-node. Hematoxylin and eosin stain, 400× original magnification, scale bar = 20 µm. Panels A, B, C, D (wtLUJV) infected.

We examined a broad array of tissue specimens collected from guinea pigs 2, 5, 7, 9, or 14 days PI and from terminal cases (Fig. 6). The most consistently observed lesions among LUJV-infected animals were hepatocyte and myocardial necrosis. No significant differences in lesion occurrence or severity were found between animals infected with wtLUJV or recLUJV.

Except in terminally ill cases, the hepatic necrotic areas were usually minute, affecting only a few (<20) hepatocytes. A mixed (heterophilic and lymphohistioplasmacytic) inflammatory infiltrate was usually admixed with necrotic hepatocytes (Fig. 6A). Generally, a pattern of multifocal hepatic necrosis and multifocal centrilobular hepatocellular macrovesiculation was observed by day 5 PI. During the course of the serial euthanasia experiment liver lesions generally became more pronounced and were classified histologically as either normal (1/15), minimal (n = 5/15), mild (n = 4/15), or moderate (3/15). Hepatic infarction was found in 3 of the 4 terminally ill animals (Fig. 6B). Despite the presence in these animals of locally extensive histologic evidence of liver necrosis and fibrin deposition, the liver enzyme activities in the sera of affected guinea pigs were consistently only moderately elevated. No significant differences were found between males and females in hepatic necrosis. The degree of hepatic necrosis was similar among non-terminal animals 5–9 days PI.

Most infected guinea pigs also developed minimal to mild myocardial necrosis with or without a histiocytic inflammatory response (Fig. 6C). Three animals developed marked lymphoplasmacytic myocarditis; 2 of these animals were terminally ill, 1 with hepatic infarction. One guinea pig incidentally had mucoid degeneration of the left atrioventricular valve (endocardiosis), usually encountered in older animals.

The kidneys of most animals were also affected. Most kidneys showed tubular degeneration, with or without mineralization and varying (usually mild) degree of interstitial lymphoplasmacytic infiltrates. Tubular degeneration was most likely due to LUJV infection. However, it is unclear if the interstitial inflammation was related to LUJV infection or was an incidental finding.

In addition to hepatic necrosis, myocarditis, and kidney pathology, the most severely affected animals developed other histologic lesions, including focal mild cortical cell necrosis in the adrenal gland, hemosiderin-laden macrophages, and erythrophagocytosis in mesenteric lymph nodes (Fig. 6D).

Generally, specimens from the lung, spleen, skeletal muscle, uterine horn or testes, adrenal gland, and urinary bladder appeared normal by routine hematoxylin and eosin histology. Other incidental findings not directly related to LUJV infection included mild to moderate focal endometritis (2 animals), and a small basal cell tumor in the stomach wall of one animal.

#### Tissue-specific gene induction

**Figure 7:**
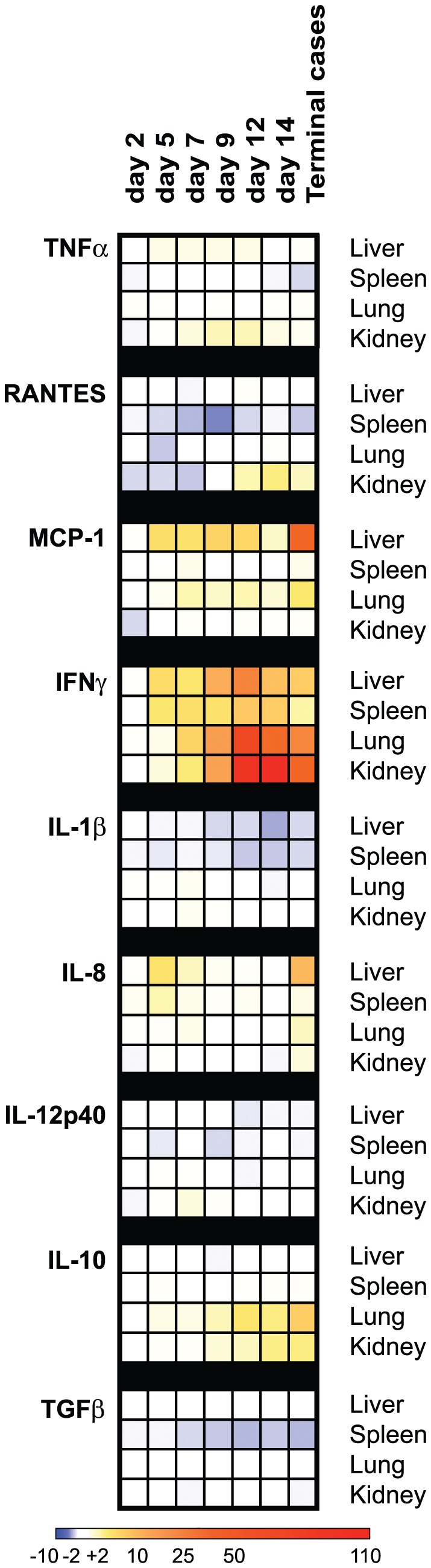
Immune mediator mRNA regulation. Heat map depicting the fold-increase or fold-decrease of mRNA from visceral tissues of infected animals compared to tissues from mock-inoculated animals. Deep blue reflects maximal fold-decrease detected (approximately −5 fold). Increasingly intensity of pale yellow to orange to red indicates range of fold-increase from +5 to a maximum of approximately +101 fold in IFNu at day 14 PI and in terminal cases. Fold-changes between −2 and +2 are depicted in white. Heat map generated with free downloadable software available from the Broad Institute (GENE-E Array, http://www.broadinstitute.org/cancer/software/GENE-E/).

Specimens of liver, lung, spleen, and kidney were collected 2, 5, 7, 9, 12, or 14 days PI, from terminal endpoint animals, and from sham-inoculated controls. Relative quantities of IL-1b, IL-2, IL-8, IL-10, IL-12p40, IFNg, MCP-1, iNOS, RANTES, TNFa, and TGFb mRNA, normalized to GAPDH, were analyzed by qRT-PCR. These were compared as groups between LUJV-infected and sham-inoculated control animals (Fig. 7 and Table S1). No significant difference in the magnitude of gene fold-changes was detected between wtLUJV and recLUJV infected animals, so all data from these animals were combined. Generally, we saw significant differences in the magnitude of gene induction across tissue types, with lung, kidney, and liver tissues all demonstrating greater fold-changes compared to spleen tissues regardless of the time PI. Broadly, we observed no change or decrease (2 to 5-fold) in several pro-inflammatory mRNA levels (IL-1b, RANTES), and modest up-regulation of TNFa (2 to 7-fold), primarily in kidneys. Most prominently, IFNg was elevated in all tissues, ranging from an approximately 15-fold increase on day 5 PI to >100 fold-increase at day 14 PI. Similarly, rises in MCP-1 gene activity were detected primarily in liver, peaking at >53-fold in terminal animals. IL-8 (a potent activator of neutrophils and other granulocytes) was upregulated (>11-fold at day 5 PI; >21 fold in terminal cases) in the liver. Only modest upregulation of IL-12p40 was detected in kidneys and lungs. Generally, we noted an induction of the anti-inflammatory/immunoregulatory gene IL-10 in all tissues, including >15-fold induction in lungs of LUJV-infected animals compared to control animals at day 14 PI (Fig. 7 and Table S1). In contrast, TGFb (another immunosuppressive mediator) was slightly reduced, but only in the spleen. Specific gene mRNA signatures for IL-2 and iNOS were not detected in any tissue at any time.

## Discussion

The pathogenic arenaviruses are genetically diverse, globally distributed, and capable of causing human illness ranging from encephalitis to severe and often lethal HF. Divided into New World and Old World lineages, these rodent-borne viruses are most often transmitted to humans by direct contact or aerosol exposure to infectious rodent excreta, or, in some cases, via a chain of human-to-human transmissions within hospital settings. The overall public health impact can range from only a few cases (e.g., Sabia or Chapare viruses) to over 100,000 cases per year (e.g., LASV) [22], [51]. LUJV is the most recently discovered pathogenic arenavirus, identified in 2008 after a high fatality (80%) cluster of cases among primary, secondary, and tertiary contacts with the index patient [52].

A number of factors mark LUJV as a unique arenavirus. Although the data from the 2008 outbreak are limited, the high case fatality was striking compared with most other arenavirus outbreaks that typically are associated with case fatalities of 10–40% [53], [54]. Phylogenetically, LUJV is distinct from all previously identified arenaviruses, forming a unique lineage more closely related to the Old World than New World arenaviruses, but with over 40% divergence from LASV at the nucleotide level [12]. The unique genomic sequence and high antigenic diversity compared to other Old World and New World arenaviruses greatly complicated its initial diagnosis by molecular (RT-PCR) or serology (IgM/IgG) techniques [11]. Unfortunately, due to these and other factors, the virus later named LUJV was only identified as the specific cause of the 2008 outbreak several weeks after the last fatal case of Lujo HF.

To assess the *in vivo* characteristics of LUJV, we began with the traditional newborn and weanling outbred mouse models of arenavirus infection [12]. These experiments further demonstrated the unique virulence properties of LUJV compared to New World (JUNV-XJ13) and Old World (LASV-Josiah) prototype arenaviruses. Both JUNV and LASV are highly virulent in newborn and weanling mice, respectively, causing lethality in an age-dependent pattern [12], [46], [47], [55]. In previous experiments, JUNV dosages as low as 1.0×10^1^ FFU caused uniform lethality after intracranial inoculation into 2-day-old mice (data not shown). Similar dosages of LASV were lethal in 14-day-old mice (data not shown). However, LUJV was non-lethal in mice regardless of the route of inoculation (intracranial, subcutaneous, or intra-peritoneal), mouse age at inoculation (2 or 14 days old), or viral dose (ranging up to the maximum dose tested, 2.0×10^3^ FFU; Fig. S1, and data not shown).

In marked contrast with the mouse results, LUJV caused severe, rapidly progressive, and uniformly lethal and hemorrhagic disease in strain 13/N guinea pigs. In this model, we observed an apparent incubation period of 5 to 6 days from the time of inoculation to the first clinical signs of illness (fever and weight loss). Over the next 24 to 48 h, the animals began to display signs of progressive illness (bilateral ocular discharge, continued fever, weight loss, and dehydration) until they were found dead or humanely euthanized when moribund. By day 5 PI, significant hematological changes began to occur, including hypoproteinemia, thrombocytopenia, and lymphopenia (Fig. 3). Interestingly, although we saw consistent elevation in key serum transaminase enzyme (aspartate transferase, alkaline phosphatase, and alanine transferase) activities, these were neither dramatic nor statistically significant and are typical of the generally poor release of tissue transaminases in guinea pigs in the face of tissue damage or necrosis. Subjective comparisons of clinical illness severity, and gross and histologic pathology between guinea pigs infected with LUJV or LASV suggests that LUJV caused a more profound illness (greater and more rapid weight loss, and frank hemorrhage and congestion in gastrointestinal organs, bladder, lymph nodes, and abdominal cavity), tissue damage (hepatic and myocardial necrosis), and hallmarks that may be consistent with disseminated intravascular coagulation (DIC) than LASV in preliminary experiments completed in our laboratory and as reported in [37] (Figs. 5 and 6, and data not shown).

Previous studies of human pathogenic New World (JUNV, Guanarito (GTOV), Machupo (MACV)) and Old World (LASV) arenaviruses using rodents and other small animal models failed to demonstrate clear consistent signs of HF (reviewed in [56]). LUJV infection of guinea pigs, however, resulted in severe infarction, fibrin deposition, and hemorrhage in multiple organs, suggesting DIC. The progressive reductions in platelet numbers (9, 12, and 14 days PI) are consistent with the consumption of platelets at sites of localized virus inflammation and tissue destruction, or, in the most extreme cases, may be consistent with DIC at later time points PI. Anecdotally, the animal with severe frank hemorrhage into the abdominal cavity developed profound thrombocytopenia (99×10^3^ uL^−1^). Unfortunately, no assays to determine coagulation factor parameters (i.e., activated partial thromboplastin time (APTT) or prothrombin time (PT)) were completed to directly assess the influence of these coagulation pathways on the observed coagulopathy. It is clear that further work is essential to definitively characterize the underlying mechanisms of these observations before firm conclusions can be drawn. For these types of studies this guinea pig model may provide a potentially useful alternative to non-human primate models for studying basic pathogenesis of a bona fide human pathogenic arenavirus causing severe coagulopathy and HF.

The rapid rise and magnitude of LUJV-specific antigen deposition, histologic pathology, and RNA titers in tissues, blood, urine, and abdominal fluid were surprising. Within 48 h of infection, the virus already disseminated to the liver, spleen, kidneys, and lungs, and was rapidly replicating up to 1.8×10^5^ TCID_50_ eq/g of liver. Interestingly, this high-titer replication continued for another 48–72 h before the onset of illness, indicated by increased body temperature and weight loss 5–6 days PI. Tissue viral loads remained extremely high throughout the course of the disease, with death occurring 11–16 days PI.

Like other arenaviruses, LUJV broadly modulates host immune responses during infection. The molecular basis of the seemingly high LUJV virulence in humans has not been characterized. However, recent work using reverse genetics-derived recombinant viruses indicates that LUJV has unique promoter elements that influence the expression of the viral NP and glycoprotein, and an unusually long intergenic region sequence on the viral L segment, which influences expression of the Z protein (Bergeron et al., in-review). Both LASV and lymphocytic choriomeningitis virus NP and Z proteins have immunomodulatory properties and function as potent antagonists of host cell antiviral responses ([21], [24], [25], [26] and reviewed in [57]). These and other yet unrecognized molecular motifs may augment the apparently enhanced virulence of LUJV by increasing viral replication or interfering with immunoregulatory mechanisms within the host.

The ability of LUJV to influence the immune response is illustrated in guinea pigs by the finding that, despite the very high viral loads by day 5 PI, pro-inflammatory cytokine/chemokine genes, such as IL-1b and RANTES, were downregulated 2–4-fold early during infection (Fig. 7). In contrast, as early as day 5 PI, potent mediators of macrophage and neutrophil activation and inflammation (IL-8, MCP-1) and pro-inflammatory molecules (IL-12p40, and IFNg) were transcriptionally upregulated (10 to >100-fold) compared to mock inoculated animals, especially in liver and kidney tissues (Fig. 7, and Table S1). Potentially important and robust induction of the broad immunomodulatory mediator IL-10 was also detected in lungs by day 9 PI, and may signal attempts to limit and control pro-inflammatory activity, allowing for further virus replication.

Overall, the pattern of gene induction and histological evidence from our study allow for the speculation that, in early stages of infection, the animals mounted a pro-inflammatory and innate immune response (presumably involving macrophages, neutrophils, NK-cells (i.e., Kurloff cells in the guinea pig), and/or NKT-cells) in multiple tissues. This was followed by a predominantly Th1 response dominated by IFNg, MCP-1, and IL-12p40, which likely stimulated activation and enhanced function presumably of NK cells, CD4+ T_H_1 cells, and/or CD8+ cytotoxic lymphocytes, resulting in a strong bias towards cell mediated immunity. Since LUJV is not highly cytopathic in cell culture, these responses may have been more deleterious than helpful to the host, due to immune cell-mediated destruction of vital organs. This hypothesis is consistent with recent work describing enhanced LASV pathogenesis due to deleterious T-cell mediated activation and stimulation of monocytes/macrophages, leading to tissue destruction in humanized HHD mice [58]. Regardless of the immune mechanisms stimulated by LUJV infection, these responses were insufficient to control virus replication and dissemination throughout the host, and to prevent eventual lethality.

Similarly to LASV infection in humans and non-human primate animal models, LUJV does not appear, at least at the mRNA transcriptional level, to elucidate an end-stage cytokine storm as seen in fatal hemorrhagic cases of infection with Ebola, Marburg, or Rift Valley fever viruses [59], [60], [61], [62]. Although IFNu-producing cells (presumably natural killer cells, NKT-cells, and/or macrophages) are clearly activated, the increase in mRNA encoding the TNFa, iNOS, and IL-1b genes was not significant even in terminal cases. Among the limited genes analyzed in this study, we speculate that complete dysregulation of the host immune response is not responsible for the dramatic vascular permeability changes and coagulopathy observed in the guinea pig model of LUJV HF. Although our results are suggestive, more definitive studies of the guinea pig immune response are necessary before drawing distinct conclusions regarding the roles of inflammatory mediators, immune effector cells, and viral virulence factors in the pathogenesis of LUJV HF in this animal model.

The dramatic severity of the clinical illness, short survival times, high tissue viral loads, and the mRNA gene expression patterns in visceral organs further highlight the unique pathogenic characteristics of LUJV compared with other studies of LASV or JUNV [37], [44]. Our *in vivo* data, taken together with recent insights into the unique genomic elements regulating viral replication in cell culture, stress the importance of further work to elucidate the possibly novel pathogenic mechanisms employed by LUJV to cause HF in both humans and guinea pigs. Direct comparisons of the precise pathogenic mechanisms utilized by LUJV and other pathogenic arenaviruses are underway, and may reveal insights into the apparent enhanced virulence of LUJV in humans and guinea pigs, and provide broader understanding of arenavirus HF. Regardless of the exact mechanisms, LUJV is clearly highly pathogenic, easily transmitted in health care settings, and a potential health threat in southern Africa. Establishing a robust and reliable animal model of severe and lethal LUJV HF is a critical first step for further investigations to increase our understanding of LUJV and for developing anti-viral therapeutics or experimental vaccines for this new and unique threat to human health.

## Supporting Information

Figure S1 **Clinical outcomes (mice).** Survival times of newborn 2-day-old (panel A) and weanling 14-day-old mice (panel B) inoculated with equivalent amounts (500 FFU) of wtLUJV, wtJUNV-XJ13, or wtLASV-Josiah intracranially. Note that no LUJV-infected or sham-inoculated animals succumbed during the 28-day monitoring period.
(EPS)

Table S1 **Gene induction data.** Fold-change of selected mRNA genes in liver, lung, spleen and kidney tissues from LUJV-infected guinea pigs over those of sham-inoculated animals. Data shown is the groupwise average, standard error of the mean (SEM) and significance. Significant deviations from normal control animal values are indicated by asterisks (p-value: *<0.05; **<0.01; ***<0.001 and ns = not significant.
(EPS)
